# Spatial Variation of Surface Soil Available Phosphorous and Its Relation with Environmental Factors in the Chaohu Lake Watershed

**DOI:** 10.3390/ijerph8083299

**Published:** 2011-08-15

**Authors:** Yongnian Gao, Junfeng Gao, Jiongfeng Chen

**Affiliations:** State Key Laboratory of Lake Science and Environment, Nanjing Institute of Geography and Limnology, Chinese Academy of Sciences, 73 East Beijing Road, Nanjing 210008, China; E-Mails: gaojunf@niglas.ac.cn (J.F.G.); woyuna@gmail.com (J.F.C.)

**Keywords:** soil available phosphorous, spatial distribution, environmental factor, Chaohu Lake watershed

## Abstract

The study presented in this paper attempts to evaluate the spatial pattern of soil available phosphorus, as well as the relation between soil available phosphorus and environment factors including elevation, slope, precipitation, percentage of cultivated land, percentage of forest land, percentage of construction land and NDVI using statistical methods and GIS spatial analysis techniques. The results showed that the Spline Tension method performed the best in the prediction of soil available phosphorus in the Chaohu Lake watershed. The spatial variation of surface soil available phosphorus was high in Chaohu Lake watershed and the upstream regions around Chaohu Lake, including the west of Chaohu lake (e.g., southwest of Feixi county, east of Shucheng county and north of Lujiang county) and to the north of Chaohu Lake (e.g., south of Hefei city, south of Feidong county, southwest of Juchao district), had the highest soil available phosphorus content. The mean and standard deviation of soil available phosphorus content gradually decreased as the elevation or slope increased. The cultivated land comprised 60.11% of the watershed and of that land 65.63% belonged to the medium to very high SAP level classes, and it played a major role in SAP availability within the watershed and a potential source of phosphorus to Chaohu Lake resulting in eutrophication. Among the land use types, paddy fields have some of the highest maximum values and variation of coefficients. Subwatershed scale soil available phosphorus was significantly affected by elevation, slope, precipitation, percentage of cultivated land and percentage of forest land and was decided by not only these environmental factors but also some other factors such as artificial phosphorus fertilizer application.

## 1. Introduction

Eutrophication, associated with discharge of nitrogen and phosphorus into water from point- and nonpoint-sources, has become a severe water pollution problem in many countries [1–3]. Chaohu Lake, one of the five largest freshwater lakes in China, is located in the center of Anhui Province, China (117.16°–117.51° E and 31.43°–31.25° N). Over the last two decades, with the population growth and the rapid economic development in the drainage area, nutrient-rich effluents have increasingly discharged into the lake [1]. This has resulted in severe contamination and eutrophication of this lake [1]. In 2008, Chaohu Lake’s water was moderately polluted, with a nutritional status of moderate eutrophication and the major pollution indicators were total phosphorus, total nitrogen and oil. The pollution was mainly from agricultural land, industrial and domestic wastewater [4]. The spatial variation analyses of surface soil available phosphorous are needed to better understand the distribution and transport of watershed nonpoint-source phosphorus across landscapes to better inform how we can manage land use activities such as precision fertilization and nutrient-based agricultural partition management to minimize environmental impacts such as lake eutrophication.

In recent decades, with the development of Geographic Information System (GIS), Global Positioning System (GPS) and Remote Sensing (RS) technology, the spatial distribution of soil available phosphorus as an important soil quality indicator has gradually become an international research focus and it has been introduced into the agricultural resources and ecological management by a variety of governments in many counties including China. Today different geostatistical and GIS spatial analysis techniques are used widely for prediction of spatial variations of the soil properties [5–8]. Robinson and Metternicht used three different techniques including Inverse Distance Weight (IDW), cokriging, and spline methods for predicting the level of soil salinity, acidity and organic matter in South West of Australia and the results showed that the cokriging and spline methods were the best techniques for estimation of the soil salinity level and organic matter content, results also showed that the IDW method was suitable for prediction of soil acidity level [5,9]. Yamashita *et al*. investigated the seasonal and spatial variation of nitrogen dynamics in the litter and surface soil layers along a slope in a tropical dry evergreen forest in northeastern Thailand [10]. Zhang *et al*. examined the spatial patterns of soil nutrient (N and P) using geostatistical analysis in three different soil horizons (A, B, C) in a mixed forest in Beijing China [11].Vaughan *et al*. quantified the spatial variation of surficial (0–5 cm) soil P within vegetated agricultural ditches on a farm in Princess Anne, MD with an approximately 30-year history of poultry litter application [12]. Corazza *et al*. studied the spatial variability of soil phosphorus content of a Rhodic Ferralsol in a low productivity pasture of *Brachiaria brizantha* (BB) grown for 10 years, without fertilizer application, in an experimental area at Planaltina (GO), Brazil [13]. Lin *et al*. evaluated the phosphorus storage and the phosphorus density of paddy soils and characterized the spatial variations of phosphorus among the subgroups of paddy soils and soil regions in China [14]. Grunwald *et al*. estimated the spatio-temporal patterns of soil total phosphorus (TP) in Water Conservation Area 2A (WCA-2A) in the Greater Everglades (GE) ecosystem and compared two different geostatistical methods: ordinary kriging (OK) and conditional sequential Gaussian simulation (CSGS), addressing spatial variability, continuity and uncertainty of TP estimations [15]. Page *et al*. investigated the soil P status, distribution, and variability, both spatially and with soil depth, of two different first-order catchments; and determinied variation in soil P concentration in relation to catchment topography (quantified as the “topographic index”) and critical source areas (CSAs) [16].

However, very little research on the spatial distribution of soil available phosphorus and total phosphorus has been conducted in the Chaohu Lake watershed [17,18]. In this paper, the spatial variation of surface soil available phosphorus and its relationship with environmental factors in the Chaohu Lake watershed were analyzed using statistical methods and GIS spatial analysis techniques.

## 2. Material and Methods

### 2.1. Study Area

**Figure 1.**
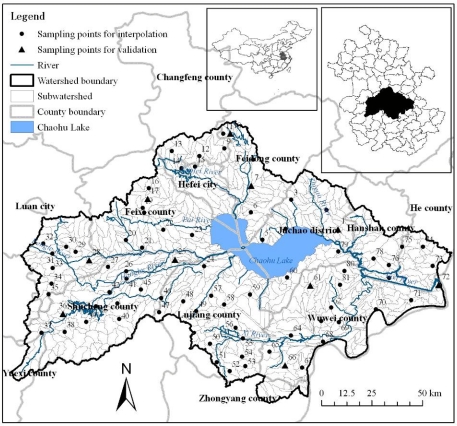
Location of the Chaohu Lake watershed in Anhui Province, China and the sampling sites of surface soil available phosphorous and subwatersheds in the Chaohu Lake watershed.

The Chaohu Lake watershed is located west of Dabie Mountain and north of the Yangtze River in the east China and central Anhui Province (Figure 1). The watershed is wide from east to west and narrow from north to south. Its area extends from 116°23′59″ to 118°22′5″ E and from 30°52′25″ to 32°7′53″ N, and the drainage area of the Chaohu Lake watershed occupies about 13,400 km^2^, with a water area of about 820 km^2^. This watershed area is a typical temperate continental monsoon climate type and belongs to subtropical and warm-temperate transitional zone, the yearly mean temperature is between 15 to 16 °C. The annual precipitation is in the 1000–1158 mm, with an average of approximately 1100 mm. Undulating terrain characterizes the topography surrounded by Fu Mountain, Yinping Mountain, and Dabie Mountain. Elevations vary from about 0 to 1,498 m with an average of around 65.76 m. The rivers in the Chaohu Lake watershed show a Chaohu Lake-centered radial distribution because of the terrain conditions. There is a high-density of river networks with a total of thirty-three rivers in the watershed, including Zhigao River, Nanfei River, Paihe River, Hangbu River, Baishishan River, Yuxi River and Zhao River.

### 2.2. Surface Soil Survey

#### 2.2.1. Sampling Sites

In this study, a total of eighty-one random soil sampling sites were set up, as shown in Figure 1. Samples were taken at depths from 0 to 0.2 m depth. Among them, there were three sampling sites in Changfeng County, five in Feidong county, two in Hefei city, eight in Feixi county, seven in Juchao district, three in Hanshan county, three in He county, fourteen in Wuwei county, fourteen in Lujiang county, fourteen in Shucheng county and eight in Lu’an city.

#### 2.2.2. Laboratory Analysis

The soil samples were taken for laboratory analysis. In laboratory, the soil samples were air-dried and passed through a 2 mm sieve before analysis, and the Olsen and Sommers method [19] was used to measure the soil available phosphorus of the eighty-one soil samples.

### 2.3. Spatial Interpolation and Validation

Interpolation is a mathematical function that estimates the values at locations where no measured values are available. In this paper, a total of six commonly used spatial interpolation methods, including the deterministic interpolation methods of regularized and tension spline, IDW, and the stochastic method of ordinary kriging with spherical, exponential, Gaussian (or normal distribution) semivariogram model, were used to predict the spatial distribution of soil available phosphorus in the Chaohu Lake watershed based on the soil samples. Spline method interpolates a surface from points using a minimum curvature spline technique, and the regularized option of Spline Type usually produces smoother surfaces than those created with the tension option. With the regularized option, higher values used for the weight parameter produce smoother surfaces. With the tension option, higher values entered for the weight parameter result in somewhat coarser surfaces, but surfaces that closely conform to the control points [20]. IDW method interpolates a surface from points using an inverse distance weighted (IDW) technique [20]. In interpolation with IDW method, a weight is attributed to the point to be measured. The amount of this weight is dependent on the distance of the point to another unknown point [5]. Kriging method interpolates a surface from points using kriging, and kriging is a processor-intensive process. When the kriging method is set to Ordinary, the available semivariogram models are spherical, circular, exponential, Gaussian, and linear [20]. And the grid cell resolution of the predicted soil available phosphorus was 90 meters. Among the eighty-one soil samples, seventy-one random samples (Figure 1) were used for the interpolation, and the remaining ten samples for model validation.

The performance of the six methods was evaluated using true validation including scatter plots and regression statistics, which involved comparison of predictions of soil available phosphorus at the remaining ten samples for model validation with their corresponding observed values. The effectiveness of each method was assessed using five indicators, including the mean of the observed variable (<O>), the mean of the model-predicted variable (<P>), the mean difference (MD), the mean absolute difference (MAD), and the mean absolute percent difference (MAPD) [21,22] as shown in Table 1, from the measured and predicted values.

### 2.4. Analysis Method of Spatial Variation

The analysis included the spatial pattern of soil available phosphorus, level delineation of soil available phosphorus content and its spatial distribution, soil available phosphorus under different elevation and slope, and soil available phosphorus under different land use. The analysis methods included the use of GIS spatial analysis and statistical analysis, and the analysis software included EXCEL, SPSS and ARCGIS. Involved data included the original soil available phosphorus content of eighty-one samples, predicted soil available phosphorus spatial data interpolated by Spline Tension method, Digital Elevation Model (DEM), slope and land use type map. The soil available phosphorus attributes were analyzed using descriptive statistical analysis by calculating the mean, maximum, minimum, range, standard deviation, coefficient of variation, and area percentage.

### 2.5. Analysis Method of Relation

Subwatersheds were used as basic calculation units for the relation analysis between soil available phosphorus and environment factors including elevation, slope, precipitation, percentage of cultivated land, percentage of forest land, percentage of construction land and NDVI. The data included DEM, precipitation, land use map and NDVI. In this paper, the 90 m SRTM DEM data was used to delineate the subwatersheds in the Chaohu Lake watershed. A watershed analysis on the terrain model for the the Chaohu Lake watershed was performed to generate data on flow direction, flow accumulation, stream definition, stream segmentation, and watershed delineation using hydrology analysis tool. After the above several processing steps and further revised according to DEM and the rivers distribution, 839 small subwatersheds (Figure 1) were obtained based on surface drainage patterns. Then the 839 subwatersheds in the Chaohu Lake watershed were selected as the basic calculation units for each factor. Based on the raster data of environmental factors and soil available phosphorus, their spatial distribution maps were produced using Zonal Statistics Tool which calculated statistics on values of each raster within the 839 subwatersheds. Then the average values of subwatershed scale soil available phosphorus and environment factors were input to SPSS software for further analysis. The relation analysis methods included the correlation analysis, one-way analysis of variance (ANOVA), multiple compare (LSD) and multiple regression analysis.

## 3. Results and Discussion

### 3.1. Validation of Interpolation Results

**Figure 2.**
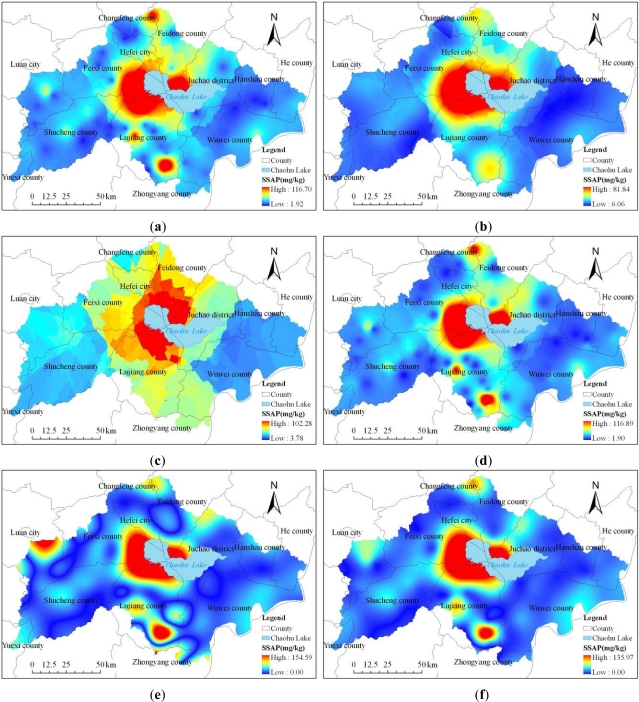
Predicted soil available phosphorus using (**a**) Kriging Ordinary Exponential (**b**) Kriging Ordinary Gaussian (**c**) Kriging Ordinary Spherical (**d**) IDW (**e**) Spline Regularized (**f**) Spline Tension methods.

Different methods have different capabilities to reflect the spatial distributions of soil available phosphorus (Figure 2). In order to quantitatively compare the differences of soil available phosphorus values predicted using the six spatial interpolation methods, a correlation analysis between the remaining ten observed and predicted values was performed and the correlation coefficients listed in Table 2. The results showed that the predicted soil available phosphorus using Spline Tension method was in best correlation with the observed values, and the correlation coefficient reached 0.906. The predicted soil available phosphorus using Kriging Ordinary Spherical method was in worst correlation with the observed values, and the correlation coefficient reached only 0.267.

**Figure 3.**
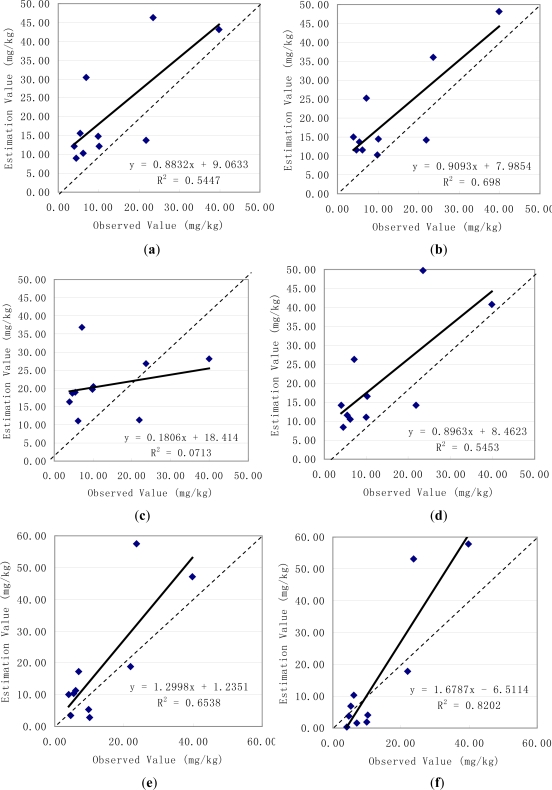
Predicted using (**a**) Kriging Ordinary Exponential (**b**) Kriging Ordinary Gaussian (**c**) Kriging Ordinary Spherical (**d**) IDW (**e**) Spline Regularized (**f**) Spline Tension methods versus observed soil available phosphorus.

Then the predicted soil available phosphorus values using the six spatial interpolation methods were plotted against the ten observed samples values, and the 1:1 lines were also plotted in the graphs (Figure 3). In Figure 3, the dashed lines corresponds to the 1:1 relation and the solid lines to the regression equations and the predicted using Spline Tension method versus observed soil available phosphorus values had the biggest decisive coefficient (R ^2^ = 0.8202). And the slope of the line in Figure 2 (f) fitted to the predicted and observed soil available phosphorus values was 1.6787 and differed from 1, and the y-intercept was −6.5114 and differed from zero, indicating that the Spline Tension method underestimated the lowest values and overestimated the highest values compared to the observed values.

For further evaluation of the differences of soil available phosphorus values estimated using the six methods, several statistics suggested by Willmott [21] and used by Timmermans *et al*.[22], listed in Table 1, were used to compare the estimation results, and the comparison between the predicted and observed soil available phosphorus values was tabulated in Table 3. From the results in Table 3, it can be seen that the mean of the observed variable was 13.25 mg/kg, while the mean of the Spline Tension model-predicted variable was 15.74 mg/kg, and their difference was only 2.49 mg/kg. The mean absolute difference (MAD) between the observed and Spline Tension method-predicted values was 8.16 mg/kg. Comparison of the values estimated by different methods in Table 3 suggests that the values of the mean of the model-predicted variable, mean difference, mean absolute difference, mean absolute percent difference increase in order as Spline Tension, Spline Regularized, Kriging Ordinary Gaussian, IDW, Kriging Ordinary Exponential and Kriging Ordinary Spherical method, respectively. This implies that Spline Tension method performed the best and then followed by the Spline Regularized method in second, Kriging Ordinary Gaussian method in third and Kriging Ordinary Spherical method the worst. So the prediction result by Spline Tension method was selected to analyze the spatial variation of surface soil available phosphorous and its relation with environmental factors in the Chaohu Lake watershed.

### 3.2. Spatial Characteristics of Surface Soil Available Phosphorus

Table 4 represents the summary statistics of soil available phosphorus of predicted watershed-scale and eighty-one soil samples. According to the statistical values, it can be found that the soil available phosphorus values observed and predicted had similar mean, minimum, maximum, range, standard deviation and variation coefficient values.

For the observed eighty-one soil samples, the values were between 1.90 and 116.88 mg/kg with an average of 18.14 mg/kg, standard deviation of 24.20 mg/kg and variation coefficient of 133.41%. For the result predicted by the Spline Tension method, the values were between 0 and 135.97 mg/kg with an average of 22.55 mg/kg, standard deviation of 26.98 mg/kg and variation coefficient of 119.65%.

**Figure 4.**
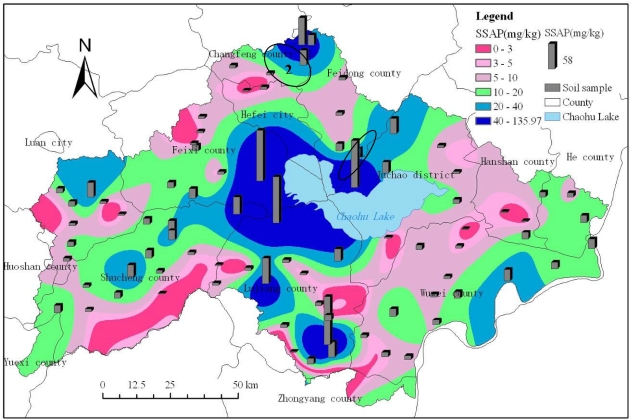
Spatial distribution of surface soil available phosphorus (SSAP) in the Chaohu Lake watershed.

According to Figure 4, it can be found that the spatial distributions of soil available phosphorus estimated using the Spline Tension method with the observed values of eighty-one soil samples were similar, and the spatial variation of surface soil available phosphorus in the Chaohu Lake watershed was high. From the spatial distribution of soil available phosphorus as exhibited in Figure 4, it can be seen that phosphorus was highest to the west of Chaohu Lake (e.g., southwest of Feixi county, east of Shucheng county and north of Lujiang county) and to the north of Chaohu Lake (e.g., south of Hefei city, south of Feidong county, southwest of Juchao district). In addition, the northernmost part of the Chaohu Lake watershed, including some area of Changfeng county and Feidong county, the west of Chaohu Lake watershed, including some area of Luan City and Shucheng county, the south of the Chaohu Lake watershed, including some area of Lujiang county and the southeast of the Chaohu Lake watershed, including some area of Wuwei county, had a relatively high soil available phosphorus values. In the other regions of the Chaohu Lake watershed, the soil available phosphorus content was relatively low.

The level delineation results of soil available phosphorus content in the Chaohu Lake watershed as shown in Table 5 showed that watershed scale soil available phosphorus content was high. Only 6.72 percent of the total watershed area belonged to the extremely low level, with an average of 1.71 mg/kg; Only 7.46 percent of the total watershed area belonged to the very low level, with an average of 4.01 mg/kg; 23.88 percent of the total watershed area belonged to the low level, with an average of 7.49 mg/kg; 30.60 percent of the total watershed area belonged to the medium level, with an average of 14.22 mg/kg; 17.16 percent of the total watershed area belonged to the high level, with an average of 27.15 mg/kg; 14.18 percent of the total watershed area belonged to the very high level, with an average of 79.96 mg/kg. The very high level had the largest standard deviation (28.75 mg/kg) and second largest variation coefficient (35.96%), the very low level had the smallest standard deviation (0.59 mg/kg) and variation coefficient (14.71%), the extremely low level had the largest variation coefficient (50.88%) and second smallest standard deviation (0.59 mg/kg).

### 3.3. Soil Available Phosphorus under Different Elevation and Slope

Based on the undulating surface condition in the Chaohu Lake watershed, it was divided into five elevation levels (Table 6) and six slope levels (Table 7). Surface elevation ranging from 0 to 10 meter belonged the level I region, 10 to 50 meter level II region, 50 to 100 meter level III region, 100 to 500 meter level IV region and 500 to 1480 meter level V region. According to the Table 6, it can be found that the soil phosphorus content in level I region ranged from 0 to 135.97 mg/kg with an average of 32.64 mg/kg, in level II region ranged from 0 to 135.97 mg/kg with an average of 21.76 mg/kg, in level III region ranged from 0 to 118.88 mg/kg with an average of 17.32 mg/kg, in level IV region ranged from 0 to 112.33 mg/kg with an average of 9.57 mg/kg, in level V region ranged from 0 to 18.16 mg/kg with an average of 9.92 mg/kg. It can also be found that the mean and standard deviation of soil available phosphorus content gradually decreased while the elevation increased. And the variation coefficient in the level IV region was largest, amounting to 129.05%, but in the level V region smallest, only 57.46%. However, the variation coefficients in level I and II regions accounting for the most of the watershed area were also large, and they reached 113.11% and 105.65% respectively. In level I and II regions, the soil available phosphorus contents were high. This is mainly because that the regions were dominated by paddy fields. And the level III region was dominated by dry land, so its soil available phosphorus content was also relative high. However, the level IV and V regions were dominated by forest land. They had lower soil available phosphorus contents because of no artificial phosphorus fertilizer application there.

Surface slope ranging from 0 to 3 degree belonged the level I region, 3 to 5 degree level II region, 5 to 8 degree level III region, 8 to 15 degree level IV region, 15 to 25 degree level V region and 25 to 49.75 degree level VI region. According to the Table 7, it can be found that the mean and standard deviation of soil available phosphorus content gradually decreased while the slope increased. And the variation coefficient in the level IV region was largest, amounting to 128.93%, but in the level VI region smallest, only 83.94%. However, the variation coefficients in level I, II, III and V regions accounting for the most of the watershed area were also large, and they reached 113.00%, 115.09%, 120.35% and 127.45% respectively. In level I region, the soil available phosphorus contents was the highest. The land use type in the area mainly consisted of cultivated land including paddy fields and dry land, and the vegetation cover was composed mainly of rice and wheat and the popular cultivation mode was rice-wheat rotation. However, the level III, IV, V and VI regions had relatively large slope. The areas were dominated mainly by forest land and had the lowest soil available phosphorus contents. Most parts of the level II region located at the junction of hills, mountains and plains, soil available phosphorus content in the area was relatively low.

### 3.4. Soil Available Phosphorus under Different Land Use

**Figure 5.**
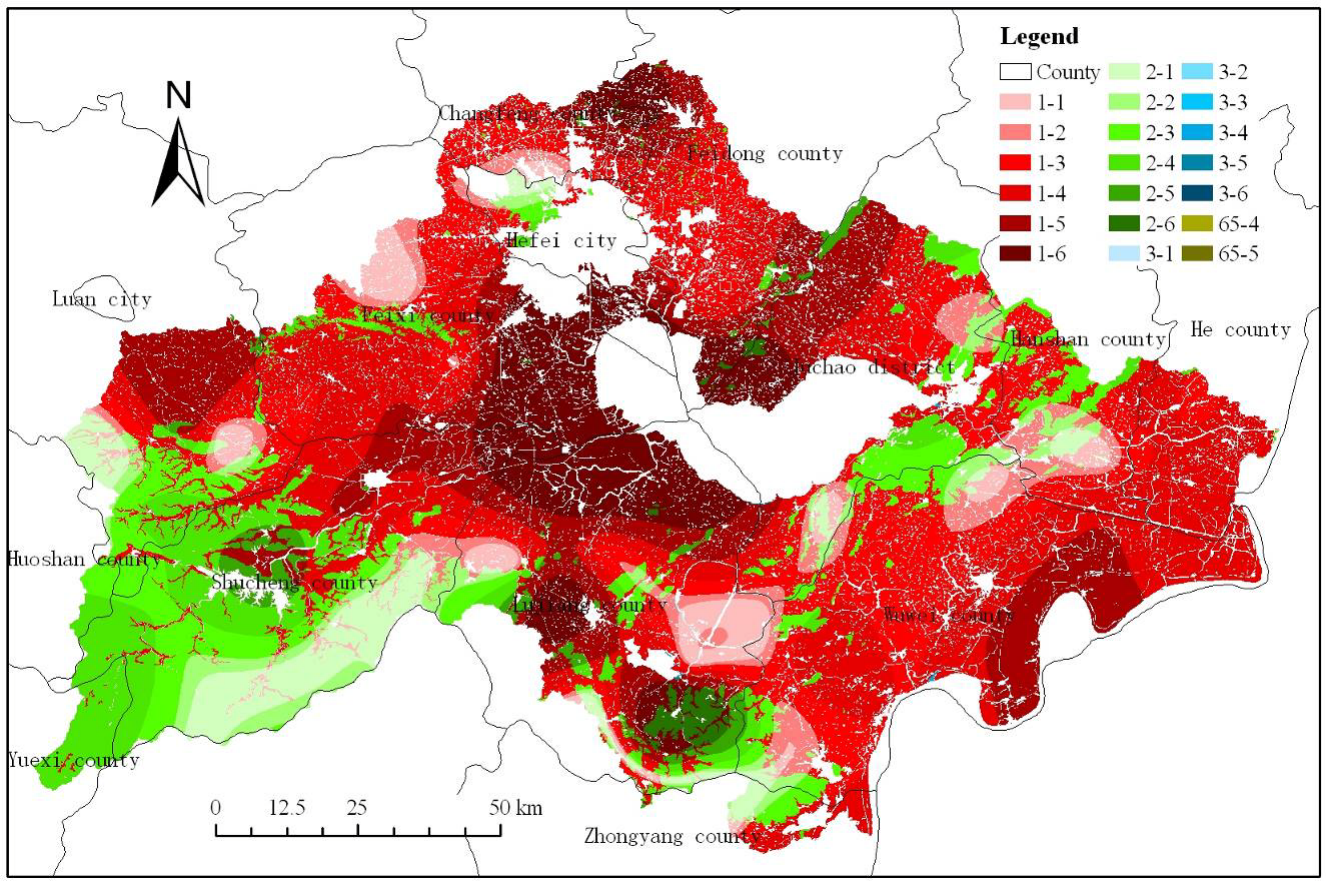
Spatial distribution of soil available phosphorus levels in different land use types. In the legend, the number before the symbol “-” represents the land use types and the 1, 2, 3 and 65 represents cultivated land, forest land, grassland, and bare land respectively, the number after the symbol “-” represents the soil available phosphorus levels as shown in Table 5.

In this paper, only the soil available phosphorus in cultivated land (including paddy fields in hilly area, paddy fields in plain area, dry land in hilly area, dry land in plain area), forest land (including dense woodland, shrub land, sparse woodland, other forest land), grassland (including high coverage grassland, medium coverage grassland) and bare land was included in the analyses. According to Table 8 and Figure 5, it can be found that the sparse woodland had the largest soil available phosphorus content with an average of 46.84 mg/kg, other forest land the second largest with an average of 31.59 mg/kg. The dense woodland had the smallest soil available phosphorus content with an average of only 10.63 mg/kg, dry land in hilly area the second smallest with an average of 11.75 mg/kg. And the paddy fields in hilly area and paddy fields in plain area, accounting for 16.14 and 32.39 percent of the watershed area respectively, had relatively larger soil available phosphorus values, and their mean values were 21.97 mg/kg and 21.97 mg/kg, respectively.

Comparison of the mean values in Table 8 suggests that the soil available phosphorus values increased in order as sparse woodland, other forest land, paddy fields in hilly area, paddy fields in plain area, bare land, medium coverage grassland, high coverage grassland, shrub land, dry land in plain area, dry land in hilly area and dense woodland, respectively. The sparse woodland consisted mainly of fruit trees such as peach, plum, apricot, jujube, pear and pomegranate trees, and the other forest land was composed mainly of other species such as poplar. The sparse woodland located in the southeast of Feidong county (No. 1 oval zone as shown in Figure 4) with the area of 387.59 hm^2^, and the other forest land located in the southeast of Changfeng county (No. 2 oval zone as shown in Figure 4) with the area of 484.93 hm^2^. These two land use types had the largest soil available phosphorus contents mainly because of long-term human fertilization. And according to the variation coefficient of soil available phosphorus in different land use types, it can be found that four kinds of land use types, including paddy fields in hilly area, paddy fields in plain area, dry land in hilly area and dense woodland, had the largest variation coefficient, we can also see that paddy fields have some of the highest maximum values and variation of coefficients. It indicates that human fertilization had a significant impact on soil available phosphorus content. Figure 5 represents the spatial distribution of soil available phosphorus levels in different land use types.

**Figure 6.**
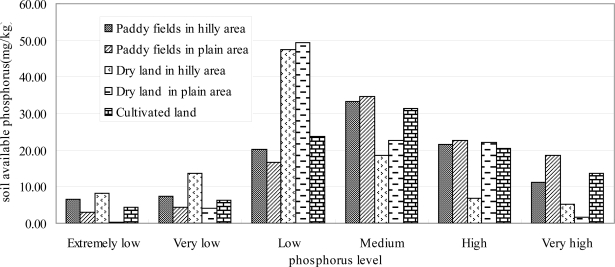
Area percentage of different soil available phosphorus levels in different cultivated land types.

According to the spatial distribution of cultivated land and soil available phosphorus (Figure 5), it can be found that only 4.35 percent of the total cultivated land area belonged to the extremely low level, only 6.27 percent the very low level, 23.74 percent the low level, 31.48 percent the medium level, 20.45 percent the high level, 13.70 percent the very high level. For the paddy fields in hilly area, 33.29 percent of the total area belonged to the medium level, 34.16 percent the extremely low and very low and low levels, 32.55 percent the high and very high levels. For the paddy fields in plain area, 34.65 percent of the total area belonged to the medium level, 24.14 percent the extremely low and very low and low levels, 41.21 percent the high and very high levels. For the dry land in hilly area, 18.62 percent of the total area belonged to the medium level, 69.14 percent the extremely low and very low and low levels, 12.24 percent the high and very high levels. For the dry land in plain area, 22.52 percent of the total area belonged to the medium level, 53.82 percent the extremely low and very low and low levels, 23.67 percent the high and very high levels (Figure 6). As a whole, cultivated land comprised 60.11% of the watershed and of that land 65.63% belonged to the medium to very high SAP level classes. It thus seems clear that cultivated land played a major role in SAP availability within the watershed and a potential source of phosphorus in Chaohu Lake, resulting in eutrophication.

### 3.5. Relation between Subwatershed Scale Soil Available Phosphorus and Environmental Factors

The correlation analysis results as shown in Table 9 indicated that the subwatershed scale soil available phosphorus (SAP) content and the various environmental factors, including average elevation (E), slope (S), precipitation (P), percentage of cultivated land (PCL_1_) and percentage of forest land (PFL), was highly significantly (*p* < 0.01) correlated. The significance of Pearson correlation coefficient between percentage of construction land (PCL_2_), NDVI and soil available phosphorus was larger than 0.05, and it showed that they did not have significant linear relationship. Correlation analysis between the different environmental factors showed that they each other also existed the highly significant correlation (Table 7). It implied that subwatershed average elevation, slope, precipitation, percentage of cultivated land and percentage of forest land had significant effects on subwatershed scale soil available phosphorus content, or these environmental factors may be affected or decided by certain other factors so that correlation relationship existed between them.

The ANOVA and LSD methods were further used to explore the relation between subwatershed scale soil available phosphorous and five significant environmental factors (Table 9) including average elevation, slope, precipitation, percentage of cultivated land and percentage of forest land and the results are shown in Table 10. From Table 10, it can be found that all the differences were significant (P < 0.05), and these showed that E, S, P, PCL_1_ and PFL had significant effects on subwatershed scale soil available phosphorus. At subwatershed scale, the mean of soil available phosphorus content gradually decreased while the elevation (except for the level 4) or slope increased, which were consistent with the above results in section 3.3. The mean of soil available phosphorus content gradually decreased (except for the level 2) while the precipitation increased. The mean of soil available phosphorus content gradually increased (except for the level 2) while the PCL_1_ increased, and SAP decreased (except for the level 4) while the PFL increased. The SAP values were high in the subwatersheds which have a large area of cultivated land or small area of forest land, for example, the mean SAP value in level 5 PCL_1_ subwatersheds was 2.31 times than that in level 1 PCL_1_ subwatersheds. And these indicated that the cultivated land had higher SAP value than forest land, which was consistent with the above results in section 3.4.

The simple Pearson correlation coefficient was calculated only taking into account the correlation between two variables, not taking into account the impact of other variables. Therefore, multiple regression analysis was further used to quantitatively measure the relation between subwatershed scale soil available phosphorus and environmental factors, and the impact of various environmental factors on the spatial patterns of subwatershed scale soil available phosphorus content. The independent variables normalized multiple regression equation was:

(1) $$\begin{array}{c} y=2.662+0.129x_{1}-0.156x_{2}-0.045x_{3}-0.012x_{4}-0.291x_{5}\\ \left(F=26.778,p<0.001,\;\text{Adjusted }R^{2}=0.133\right) \end{array}$$

where *y* was the natural logarithm of subwatershed scale soil available phosphorus content, *x*_1_ was the normalized value of elevation, *x*_2_ was the normalized value of slope, *x*_3_ was the normalized value of precipitation, *x*_4_ was the normalized value of percentage of cultivated land, and *x*_5_ was the normalized value of percentage of forest land. Regression equation (1) showed that although it reached a significant level (*p* < 0.01), the explained variance of the impact of these five factors on soil available phosphorus was only 13.3 percent, it indicated that the subwatershed scale soil available phosphorus was decided not only by these environmental factors but also by some other factors such as artificial phosphorus fertilizer application, and the relation between SAP and these five environmental factors was complex and maybe non linear and can’t be expressed by a simple linear equation in the Chaohu Lake watershed.

## 4. Conclusions

Based on the study on spatial variation of surface soil available phosphorous and its relation with environmental factors in the Chaohu Lake watershed, the following four conclusions were obtained:

1. In the prediction of soil available phosphorus in the Chaohu Lake watershed, the Spline Tension method performed the best and then followed by the Spline Regularized method in second, Kriging Ordinary Gaussian method in third and Kriging Ordinary Spherical method the worst.
2. The spatial variation of surface soil available phosphorus was high in the Chaohu Lake watershed. The upstream regions around Chaohu Lake, including the west of Chaohu Lake (e.g., southwest of Feixi county, east of Shucheng county and north of Lujiang county) and to the north of Chaohu Lake (e.g., south of Hefei city, south of Feidong county, southwest of Juchao district), had the highest soil available phosphorus content.
3. The mean and standard deviation of soil available phosphorus content gradually decreased as the elevation or slope increased. The regions ranging from 0 to 50 meters of elevation were mainly dominated by paddy fields and had the highest soil available phosphorus contents, the regions ranging from 100 to 1480 meters of elevation were mainly dominated by forest land and had the lowest soil available phosphorus contents. The region ranging from 0 to 3 degrees of slope was dominated by cultivated land and had the highest soil available phosphorus content, the regions ranging from 5 to 49.75 degrees of slope were dominated by forest land and had the lowest soil available phosphorus contents. The cultivated land comprised 60.11% of the watershed and of that land 65.63% belonged to the medium to very high SAP level classes, and it played a major role in SAP availability within the watershed and a potential source of phosphorus to Chaohu Lake, resulting in eutrophication. Among the land use types, paddy fields have some of the highest maximum values and variation of coefficients.
4. The subwatershed scale soil available phosphorus content and the various environmental factors, including average elevation, slope, precipitation, percentage of cultivated land and percentage of forest land, were significantly correlated, the percentage of construction land, NDVI and soil available phosphorus did not have significant linear relationship. The results after ANOVA and LSD analyses between subwatershed scale soil available phosphorous and five significant environmental factors showed that all the differences were significant (P < 0.05), and E, S, P, PCL_1_ and PFL had significant effects on subwatershed scale soil available phosphorus. Subwatershed scale soil available phosphorus was decided not only by these environmental factors but also by some other factors such as artificial phosphorus fertilizer application.

## Figures and Tables

**Table 1.** Description of the statistics used in assessing the model performance.

**Statistical variable**	**Description**	**Equation**
<*O*>	Mean of the observed variable	$\frac{1}{n}\sum_{i=1}^{n}O_{i}$
<*P*>	Mean of the model-predicted variable	$\frac{1}{n}\sum_{i=1}^{n}P_{i}$
MD	Mean difference	$\frac{1}{n}\sum_{i=1}^{n}\left(P_{i}-O_{i}\right)$
MAD	Mean absolute difference	$\frac{1}{n}\sum_{i=1}^{n}\left|P_{i}-O_{i}\right|$
MAPD	Mean absolute percent difference	$\frac{100}{<O>}\left(\frac{1}{n}\sum_{i=1}^{n}\left|P_{i}-O_{i}\right|\right)$

*n* represents the number of observations, and *n* = 10.

**Table 2.** Pearson correlation coefficient and statistical significance between the observed and predicted soil available phosphorus values using different interpolation methods.

**Method**	**Kriging Ordinary Exponential**	**Kriging Ordinary Gaussian**	**Kriging Ordinary Spherical**	**IDW**	**Spline Regularized**	**Spline Tension**
**Pearson correlation coefficient**	0.738*	0.835**	0.267	0.738*	0.809**	0.906**
**Significance (Two-tailed)**	0.015	0.003	0.456	0.015	0.005	0.000

** Correlation is significant at the 0.01 level (Two-tailed);

* Correlation is significant at the 0.05 level (Two-tailed).

**Table 3.** Statistics of model performance for predicting soil available phosphorus.

**Method**	**<*O*>(mg/kg)**	**<*P*>(mg/kg)**	**MD(mg/kg)**	**MAD(mg/kg)**	**MAPD(%)**
**Kriging Ordinary Exponential**	13.25	20.77	7.52	9.12	68.78
**Kriging Ordinary Gaussian**	13.25	20.04	6.78	8.32	62.80
**Kriging Ordinary Spherical**	13.25	20.81	7.55	12.05	90.92
**IDW**	13.25	20.34	7.09	8.62	65.04
**Spline Regularized**	13.25	18.46	5.21	8.32	62.77
**Spline Tension**	13.25	15.74	2.48	8.16	61.56

**Table 4.** Summary statistics of soil available phosphorus of soil samples and predicted watershed-scale.

**Item**	**Mean (mg/kg)**	**Minimum (mg/kg)**	**Maximum (mg/kg)**	**Range (mg/kg)**	**Std. Deviation(mg/kg)**	**Variation coefficient(%)**
**Soil samples**	18.14	1.90	116.88	114.98	24.20	133.41
**Watershed scale**	22.55	0.00	135.97	135.97	26.98	119.65

**Table 5.** Summary statistics of soil available phosphorus of different nutrient levels.

**Level code**	Level range(mg/kg)^*^	**Level description**	**Area percentage(%)**	**Mean (mg/kg)**	**Std. Deviation(mg/kg)**	**Variation coefficient(%)**
1	<3	Extremely low	6.72	1.71	0.87	50.88
2	3–5	Very low	7.46	4.01	0.59	14.71
3	5–10	Low	23.88	7.49	1.45	19.36
4	10–20	Medium	30.60	14.22	2.84	19.97
5	20–40	High	17.16	27.15	5.50	20.26
6	>40	Very high	14.18	79.96	28.75	35.96

* The level was delineated according to the classification standard of soil fertility of second soil survey of China [23].

**Table 6.** Summary statistics of soil available phosphorus of different elevation levels.

**Elevation level code**	**Elevation range (m)**	**Area percentage (%)**	**Minimum (mg/kg)**	**Maximum (mg/kg)**	**Mean (mg/kg)**	**Std. Deviation (mg/kg)**	**Variation coefficient (%)**
1	0–10	28.57	0.00	135.97	32.64	36.92	113.11
2	10–50	43.61	0.00	135.97	21.76	22.99	105.65
3	50–100	13.53	0.00	118.88	17.32	15.25	88.05
4	100–500	12.03	0.00	112.33	9.57	12.35	129.05
5	500–1480	2.26	0.00	18.16	9.92	5.70	57.46

**Table 7.** Summary statistics of soil available phosphorus of different slope levels.

**Slope level code**	**Slope range (degree)**	**Area percentage (%)**	**Minimum (mg/kg)**	**Maximum (mg/kg)**	**Mean (mg/kg)**	**Std. Deviation (mg/kg)**	**Variation coefficient (%)**
1	0–3	78.47	0.00	135.97	25.46	28.77	113.00
2	3–5	4.77	0.00	135.52	15.23	17.52	115.09
3	5–8	3.76	0.00	133.17	13.17	15.85	120.35
4	8–15	6.25	0.00	119.53	11.58	14.94	128.93
5	15–25	5.21	0.00	119.43	9.53	12.15	127.45
6	25–49.75	1.54	0.00	106.53	8.41	7.06	83.94

**Table 8.** Summary statistics of soil available phosphorus of different land use types.

Land use type code*	**Area percentage (%)**	**Minimum (mg/kg)**	**Maximum (mg/kg)**	**Range (mg/kg)**	**Mean (mg/kg)**	**Std. Deviation (mg/kg)**	**Variation coefficient (%)**
112	16.14	0.00	130.75	130.75	21.97	25.27	115.02
113	32.39	0.00	135.97	135.97	21.97	28.03	127.58
122	7.14	0.00	100.22	100.22	11.75	12.88	109.62
123	4.44	1.32	96.27	94.95	13.94	10.35	74.25
21	19.60	0.00	130.67	130.67	10.63	12.95	121.83
22	2.06	0.00	108.13	108.13	15.75	11.99	76.13
23	0.03	22.71	85.02	62.32	46.84	27.37	58.43
24	0.03	5.28	57.54	52.26	31.59	14.59	46.19
31	0.03	0.03	104.81	104.78	17.45	17.06	97.77
32	0.01	10.43	32.07	21.65	18.44	9.08	49.24
65	0.001	19.95	23.18	3.23	21.51	1.23	5.72

* *Notes*: 112—Paddy fields in hilly area, 113—Paddy fields in plain area, 122—Dry land in hilly area, 123—Dry land in plain area, 21—Dense woodland, 22—Shrub land, 23—Sparse woodland, 24—Other forest land, 31—High coverage grassland, 32—Medium coverage grassland, 65—Bare land.

**Table 9.** Correlation coefficient of subwatershed scale soil available phosphorus content and environmental factors.

	**SAP**	**E**	**S**	**P**	**PCL_1_**	**PFL**	**PCL_2_**	**NDVI**
**SAP**	1	−0.180^**^	−0.216^**^	−0.192^**^	0.213^**^	−0.256^**^	0.008	−0.021
**E**	−0.180^**^	1	0.898^**^	0.345^**^	−0.640^**^	0.747^**^	−0.177^**^	0.568^**^
**S**	−0.216^**^	0.898^**^	1	0.393^**^	−0.748^**^	0.872^**^	−0.202^**^	0.630^**^
**P**	−0.192^**^	0.345^**^	0.393^**^	1	−0.195^**^	0.386^**^	−0.430^**^	0.401^**^
**PCL_1_**	0.213^**^	−0.640^**^	−0.748^**^	−0.195^**^	1	−0.863^**^	−0.108^**^	−0.252^**^
**PFL**	−0.256^**^	0.747^**^	0.872^**^	0.386^**^	−0.863^**^	1	−0.248^**^	0.580^**^
**PCL_2_**	0.008	−0.177^**^	−0.202^**^	−0.430^**^	−0.108^**^	−0.248^**^	1	−0.463^**^
**NDVI**	−0.021	0.568^**^	0.630^**^	0.401^**^	−0.252^**^	0.580^**^	−0.463^**^	1

** Correlation is significant at the 0.01 level (Two-tailed).

**Table 10.** Effect of different environment factors on soil available phosphorus in subwatershed scale (mg/kg).

**level**	**E**	**S**	**P**	**PCL_1_**	**PFL**
**1**	29.64a	24.63a	23.76a	11.38a	27.11a
**2**	22.48b	15.38b	33.49b	15.64ab	20.00b
**3**	16.46c	12.41b	19.07ac	13.22a	14.38bc
**4**	9.63d	9.70b	15.31c	22.72bc	11.11c
**5**	10.86bcde	8.26b	10.70c	26.27c	11.16c
***F* value**	12.049*	10.749*	18.253*	12.098*	18.033*
***Sig.***	0.000	0.000	0.000	0.000	0.000

* The difference is significant (P < 0.05): values in each column with the same letter are not significantly different among environment factors. The five levels for P was <1100 mm, 1100–1200 mm, 1200–1300 mm, 1300–1400 mm and >1400 mm, the five levels for PCL_1_ was <20%, 20–40%, 40–60%, 60–80% and >80%, the five levels for PFL was <5%, 5–15%, 15–25%, 25–55% and >55%.
